# Demographic and Clinical Spectrum of Forensic Cases in the Emergency Department: A Retrospective Analysis of 25 887 Presentations

**DOI:** 10.5152/eurasianjmed.2026.261626

**Published:** 2026-10-19

**Authors:** Fatma Tortum, Erdal Tekin, Ahmet Nezih Kök

**Affiliations:** 1 Department of Emergency Medicine, Atatürk University Faculty of Medicine, Erzurum, Türkiye; 2 Department of Forensic Medicine, Atatürk University Faculty of Medicine, Erzurum, Türkiye

**Keywords:** Emergency department, forensic presentations, injury mechanisms, pediatric presentations, predictors of mortality, seasonal variation

## Abstract

**Background:**

This study aims to evaluate the demographic and temporal characteristics of forensic presentations involving physical or psychological harm, negligence, or intentional injury and to analyze the specific factors influencing mortality among these presentations.

**Methods:**

This single-center, retrospective, cross-sectional study was conducted on forensic presentations presenting to the emergency department. Demographic data, injury mechanisms, seasonal variations, and clinical outcomes were evaluated. Data retrieved from the hospital’s electronic health record system were analyzed statistically. Multivariable logistic regression analysis was performed to identify independent predictors of mortality.

**Results:**

A total of 25887 presentations were included in the study. Of the participants, 79.8% were adults and 68.9% were male, with a mean age of 34.0 ± 18.8 years. Motor vehicle accidents (21.0%) and falls from height (17.2%) were the most common injury mechanisms. Forensic presentations peaked in August (11.1%). While discharge rates were significantly higher in adults (61.9% vs. 41.3%; *P* < .001), mortality was significantly higher in pediatrics (2.1% vs. 1.2%; *P* < .001). Multivariable regression analysis identified firearm injuries (adjusted odds ratio = 7.12) as the mechanism most strongly associated with mortality in the adjusted model of mortality.

**Conclusion:**

Forensic presentations to the emergency department showed a significant concentration among young adult males and during the summer months. Although motor vehicle accidents and falls from height were the most frequent etiologies, multivariable analysis identified firearm injuries as the mechanism most strongly associated with mortality in the adjusted model of mortality. These findings suggest that the findings may help inform triage and resource-planning studies for high-risk injury mechanisms and pediatric populations. Furthermore, healthcare facilities should implement strategic resource and personnel planning to account for the seasonal surges in forensic presentations.

**Main Points**

- This study evaluated the demographic and temporal characteristics of forensic presentations involving physical or psychological harm, neglect, or intentional injury and analyzed specific factors affecting mortality.
- This study included 25 887 presentations, 21.0% of whom had motor vehicle accidents.
- In the study, mortality rates were significantly higher in pediatric presentations.
- Multivariate regression analysis identified gunshot wounds as the mechanism most strongly associated with mortality in the adjusted model of death.

## Introduction

Forensic presentations encompass any condition that may impair an individual’s physical or mental health, result in mortality, or arise from intentional acts, negligence, or recklessness by the individual or third parties.^1^ Globally, emergency departments (EDs) serve as the frontline for such presentations, ranging from motor vehicle accidents and fall from height to interpersonal violence, poisonings, and environmental injuries like electrocution. According to the World Health Organization (WHO), injuries and violence account for a significant portion of the global burden of disease, necessitating a multidisciplinary approach in acute care settings.^2^

In many jurisdictions, including Türkiye, the reporting of forensic presentations is not merely a clinical preference but a legal mandate. Under Article 280 of the Turkish Penal Code, healthcare professionals are legally obligated to report suspected crimes encountered during their duties.^3^ This aligns with international medical–legal standards where the duty to report often supersedes patient confidentiality in presentations of public interest or criminal suspicion.

However, the identification and management of these presentations in the ED present a complex challenge for emergency physicians.^4^ While the primary objective is the stabilization of vital functions, physicians must simultaneously act as forensic examiners—identifying, documenting, and reporting incidents regardless of the patient’s role as a perpetrator or victim, or their consent.^5^ International literature suggests that a lack of standardized forensic training among ED staff often leads to under-reporting or inadequate documentation, which can hinder judicial processes.^6^ Furthermore, patients or relatives may intentionally withhold information to avoid legal repercussions, making the physician’s diagnostic suspicion and regional epidemiological knowledge crucial.

Understanding the regional characteristics, frequency, and demographic distribution of forensic presentations is essential for optimizing ED resources and improving the quality of forensic reporting.^7^ While several studies have examined forensic patterns in various global regions, local epidemiological data remain vital for tailoring regional health policies. This study aims to analyze the descriptive data of forensic presentations presenting to a tertiary hospital in Erzurum, Türkiye and to contextualize these findings within the broader international literature to facilitate more accurate identification and management of forensic presentations in the emergency setting.

## Material and Methods

### Study Design and Population

This study was conducted as a single-center, retrospective cross-sectional study in a tertiary ED. This center, where the study was conducted, provides healthcare services to 12 cities around it and approximately 4.5 million people. The study has been conducted in accordance with the principles set forth in the Helsinki Declaration as revised in 2024. Ethical approval for the study was obtained from the Clinical Research Ethics Committee of Atatürk University (date: October 25, 2026, decision no: 7/24). Informed consent requirement was waived due to the retrospective nature of the study.

Among presentations who presented to the ED of this hospital between January 1, 2014, and December 31, 2024, those who were recorded in the hospital information management system as forensic presentations and whose electronic files were accessible were included. Presentations that were not evaluated as forensic or whose necessary data could not be accessed from electronic files were excluded. Furthermore, occupational accidents were excluded because their specific mechanisms (such as motor vehicle accidents, falls from height, assault, poisoning, electrocution, or lightning strikes) were not clearly specified in the reasons for admission.

For the study, presentations age, gender, date of ED admission, presenting complaint, final diagnosis in the ED, ED outcome (death in the ED, hospitalization, or discharge), and clinical outcome (death or discharge) were obtained from electronic files.

For the period between January 1, 2014, and January 1, 2024, a total of 56 974 forensic presentations were identified in the hospital information management system. Following a rigorous data cleaning process, 31 087 presentations were excluded: 12 875 due to incorrect forensic coding, 9477 due to missing clinical outcome data, and 8735 due to incomplete presentation details. Consequently, a final study of 25 887 forensic presentations met the inclusion criteria and were included in the final analysis (Figure 1).

### Statistical Analysis

Data were analyzed using the Statistical Package for the Social Sciences version 25 (IBM Corp., Armonk, NY). The normality of continuous variables was evaluated using the Kolmogorov–Smirnov test. Descriptive statistics are reported as frequencies and percentages for categorical variables, while continuous variables are expressed as medians with interquartile ranges due to non-normal distribution. Group comparisons were performed using the Student’s *t*-test for parametric data and the Mann–Whitney *U-*test for non-parametric data.

To identify independent predictors of clinical outcomes (death and discharge), a multivariable logistic regression analysis was performed. Variables that showed a significant association in the univariate analysis (*P* < .10) were included in the model. Results were presented as odds ratios (OR) with corresponding 95% CI. Model fit was assessed using the Hosmer–Lemeshow goodness-of-fit test. A *P*-value of < .05 was considered statistically significant for the final model. The authors declared that no Artificial Intelligence Tool was used in the preparation of the manuscript.

## Results

A total of 25 887 presentations met the formal inclusion criteria and were enrolled in the study. The study group consisted mostly of adults (79.8%, n = 20 664), with an overall mean age of 34.0 ± 18.8 years. Regarding gender distribution, males constituted the majority of presentations (68.9%), whereas females accounted for 31.1% (n = 8059) of the presentations. The analysis of injury mechanisms revealed that motor vehicle accidents were the leading etiology for forensic ED admissions, representing 21.0% of the total volume. Falls from height were the second most prevalent mechanism, observed in 17.2% of presentations, followed by interpersonal violence (assault) at 12.7%. Conversely, incidents involving sexual abuse were the least frequent, comprising only 0.3% of the forensic presentations. A comprehensive breakdown of admission etiologies and demographic characteristics is presented in Table 1.

Analysis of the temporal distribution revealed significant monthly variations in forensic presentations. The peak incidence of forensic admissions occurred during the summer months, with August reaching the highest frequency at 11.1%, followed closely by July (10.8%). Conversely, the lowest volume of forensic presentations was recorded in February (6.0%). The comprehensive monthly distribution of forensic admissions is illustrated in Figure 2. When examining the distribution of forensic presentations by year of admission, it was found that the highest number of admissions occurred in 2024 (n = 3567, 13.8%), whereas the lowest was recorded in 2014 (n = 1847, 7.1%) (Figure 3).

Significant age-related disparities were observed in presentations disposition and clinical outcomes. The rate of discharge from the ED was significantly higher among adults (≥18 years) compared to the pediatric population (<18 years) (61.9% vs. 41.3%, respectively; *P* < .001). Conversely, pediatrics exhibited a significantly higher mortality rate (2.1%) compared to their adult counterparts (*P* < .001). Detailed treatment modalities and comparative clinical outcome rates stratified by age group are summarized in Table 2.

Multivariable logistic regression identified several independent predictors of mortality (Hosmer–Lemeshow *P* = .684). Among injury mechanisms, firearm injuries (adjusted odds ratios [AOR] = 7.12; 95% CI = 3.80-11.90) and carbon monoxide poisoning (AOR = 4.85; 95% CI = 2.95-7.60) carried the highest risk (*P* < .001). Other significant predictors included burns (AOR = 3.15), falls from height (AOR = 2.90), and stabbings (AOR = 1.95). Demographically, each 1-year increase in age raised mortality risk by 4% (AOR = 1.04, *P* < .001), while male gender was not an independent predictor (*P* = .210). Presentations requiring inpatient treatment had a 4.65-fold higher risk of death compared to those discharged from the ED (*P* < .001). Detailed ORs, 95% CIs, and significance levels for all injury mechanisms and other clinical variables are presented in Table 3.

## Discussion

This retrospective cross-sectional study, encompassing a large study of over 25 000 presentations, elucidates the comprehensive demographic and clinical spectrum of forensic presentations in the ED. The authors’ findings highlight 3 critical aspects of forensic presentation management: the significant impact of seasonal variations, the heightened mortality risk in pediatric populations compared to adults, and the profound lethality of firearm-related injuries. While the existing literature frequently examines forensic presentations within specific mechanistic subgroups, a holistic evaluation of the entire forensic spectrum is essential for a thorough understanding of epidemiological profiles and clinical prognoses.^8^^,^^9^ By integrating diverse injury mechanisms into a single comprehensive analysis, this study contributes to the limited body of large-scale research and provides a broader perspective on the systemic management of forensic emergencies.

In this study, the predominance of young adult males (mean age: 34.0 ± 18.8 years; 68.9% male) among forensic ED presentations aligns with international trauma literature.^10^ Similar demographic trends have been reported by Kacmaz et al^7^ and Cetin et al^11^, who emphasized the prevalence of male and young adult populations in forensic reporting. However, a significant finding in this study was the temporal fluctuation of forensic presentations, which reached a peak in August (11.1%). This summertime surge is likely associated with increased mobility, outdoor activities, and heightened behavioral aggression. This observation is consistent with the research conducted in London, which linked seasonal fluctuations in crime rates to increased impulsive behaviors during warmer months.^12^ These findings provide compelling evidence for the necessity of seasonally adjusted ED resource management. Given the sharp contrast between the minimum presentation volume in February and the peak in August, implementing season-specific staffing models and trauma team allocations appears to be a more rational and cost-effective strategy for optimizing ED workflows.

The general trauma literature focuses on increased mortality in traumas associated with forensic presentations in geriatric patients due to comorbidities in the elderly population.^13^^-^^15^ In contrast to existing literature, this study revealed a significantly higher mortality rate among pediatric patients compared to adults (2.1% vs. 1.2%, respectively). Furthermore, the discharge rate was notably lower in the pediatric group (41.3%) than in the adult population (61.9%). These findings diverge from previous reports on pediatric forensic presentations, where mortality rates typically range between 0.015% and 0.4%.^16^^-^^18^ This discrepancy represents one of the most striking results of this study, underscoring the unexpected severity of pediatric forensic presentations and the necessity for an aggressive clinical approach in this vulnerable population. While literature often emphasizes geriatric mortality due to comorbidities, the under-18 and over-18 categorization highlights a distinct risk profile. The elevated pediatric mortality may be attributed to limited physiological reserves and a unique hemodynamic response; unlike adults, pediatric presentations can remain normotensive despite losing up to 40% of their total blood volume through robust systemic vascular resistance and a potent catecholamine surge. However, this aggressive compensation often masks the clinical severity, potentially delaying recognition until the onset of irreversible decompensation.^19^^,^^20^ This pathophysiological masking effect likely explains the disproportionately high mortality observed in this pediatric study compared to adults.

In this study, motor vehicle accidents emerged as the leading etiology of forensic ED visits at 21.0%. This result is consistent with the established forensic epidemiology in the authors’ region, reflecting the high prevalence of transportation-related injuries.^18^^,^^21^^,^^22^ Following motor vehicle accidents, falls from height and physical assaults constituted the other most prevalent forensic categories. Together, these groups account for approximately half of the total presentation distribution and are predominantly characterized by blunt trauma. Blunt trauma involves energy transfer across a broad surface area, which can lead to multi-organ injuries, occult hemorrhages, and complex fractures.^10^^,^^23^ Although these presentations constitute the majority of presentations, the multivariable logistic regression analysis revealed that blunt trauma-related mechanisms carry a lower mortality risk compared to firearm injuries. Despite being less frequent than blunt trauma in the study, firearm injuries exhibited a significantly higher fatality rate (AOR: 7.12) and emerged as the mechanism most strongly associated with mortality in the adjusted model of mortality. This is attributed to the high-energy tissue cavitation and uncontrollable massive internal hemorrhaging characteristic of firearm injuries. These findings reinforce the clinical significance of the widely recognized “Golden Hour” approach and support the implementation of aggressive, immediate surgical strategies to improve presentations prognosis.^10^^,^^24^ Furthermore, the finding that male sex was not a statistically significant predictor suggests that mortality risk is more directly associated with injury severity and the mechanism of exposure rather than gender variables. Finally, the decision to hospitalize was found to increase the risk of mortality by 4.65-fold, indicating that the clinical decision for inpatient admission should itself be recognized as a significant prognostic indicator.

### Limitations

This study has several limitations. First, its retrospective design made it dependent on the accuracy and availability of hospital electronic health records, which resulted in the exclusion of detailed medical histories, such as comorbidities and chronic medication use. Furthermore, due to the retrospective design of the study and the nature of the database infrastructure, it was not possible to track recurrent admissions or presentations. Therefore, analyses were performed on an admission basis rather than a patient-by-patient basis, and each clinical admission was considered an independent event. Similarly, due to the retrospective and database-based design of the study, a significant portion (54.6%) of the total 56 974 forensic presentations were excluded due to incorrect forensic coding, missing clinical outcomes, and incomplete admission data. Since it cannot be assumed that the missing data were entirely random, deficiencies in records in specific subgroups involving fatal presentations, referrals to other centers, or complex trauma may have led to potential selection bias in both etiological distribution and mortality estimation. Due to the retrospective nature of the database and record limitations, detailed clinical profiles of the excluded presentations could not be accessed and compared with the included cohort. While this limits the generalizability of this study’s findings to the general population, the authors’ analyses were performed only on presentations for which reliable and complete data were available. Second, this was a single-center study conducted on a specific study, which may limit the generalizability of the findings to different socioeconomic or geographic regions. Third, the authors focused exclusively on in-hospital mortality; therefore, pre-hospital fatalities or deaths occurring post-discharge due to delayed complications were not captured. Furthermore, although the logistic regression model identified significant predictors such as age and injury mechanism, the modifying effects of underlying comorbidities or medications on patient outcomes could not be fully standardized due to missing data. However, the high goodness-of-fit demonstrated by the Hosmer–Lemeshow test confirms that despite these limitations, the dataset remains a robust and reliable guide for clinical decision-making.

This retrospective study investigated the demographic characteristics, temporal variations, and clinical spectra of all forensic ED presentations, regardless of age or case type. The authors’ analysis reveals a significant concentration of forensic presentations among young adult males, with a distinct seasonal peak during the summer months. Although motor vehicle accidents and falls from height were the most frequent etiologies, multivariable analysis identified firearm injuries as the mechanism most strongly associated with mortality in the adjusted model of mortality. Furthermore, the pediatric population exhibited significantly higher mortality and lower discharge rates compared to adults. These findings underscore the urgent need for specialized clinical algorithms targeting high-risk mechanisms, such as firearm injuries, and vulnerable age groups. Additionally, healthcare facilities should implement strategic resource and personnel planning to effectively manage the predictable seasonal surges in forensic presentations.

## Figures and Tables

**Figure 1.**
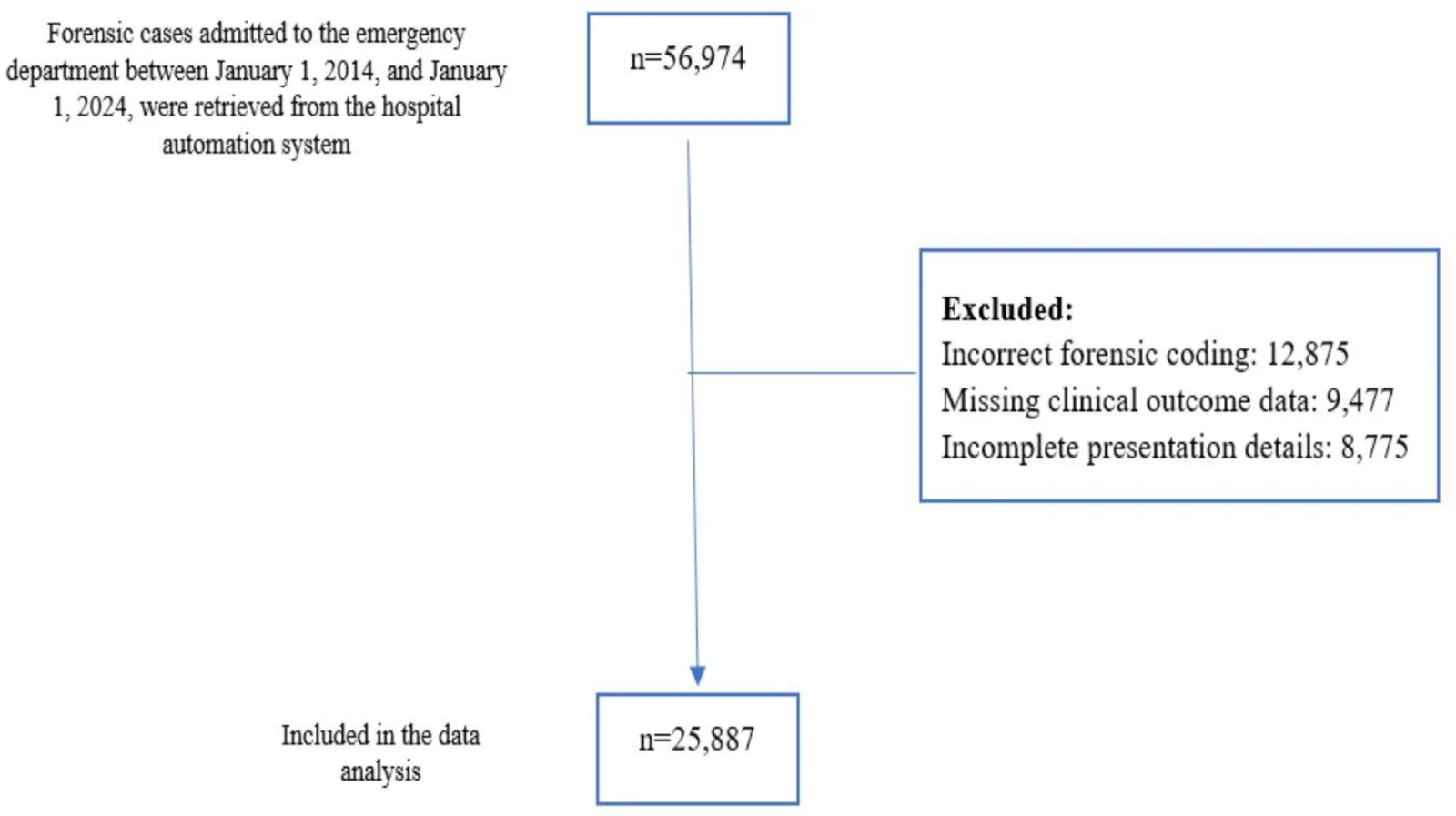
Inclusion and exclusion flowchart of the study.

**Figure 2.**
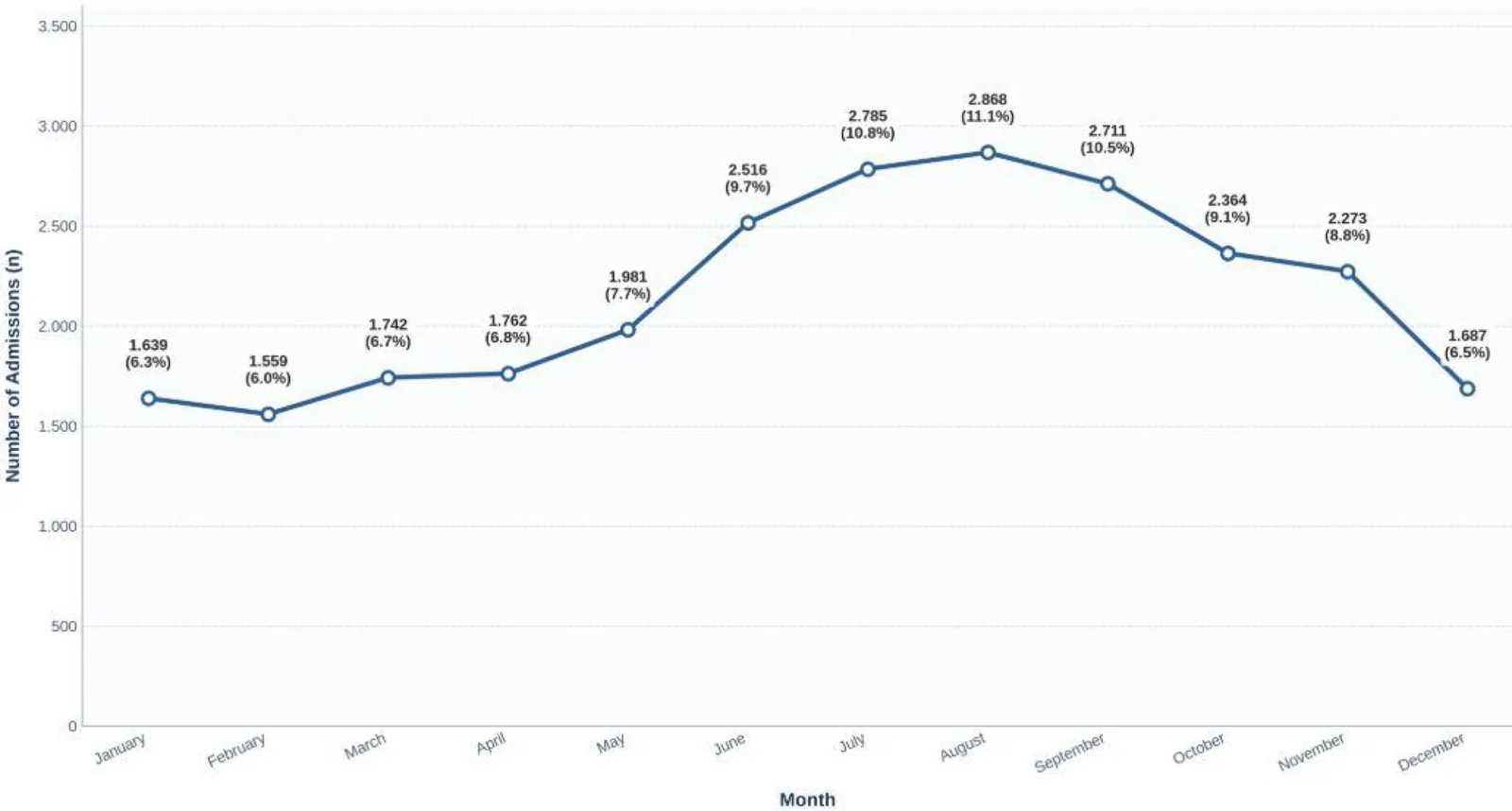
Distribution of forensic case presentations admitted to the emergency department by months.

**Figure 3.**
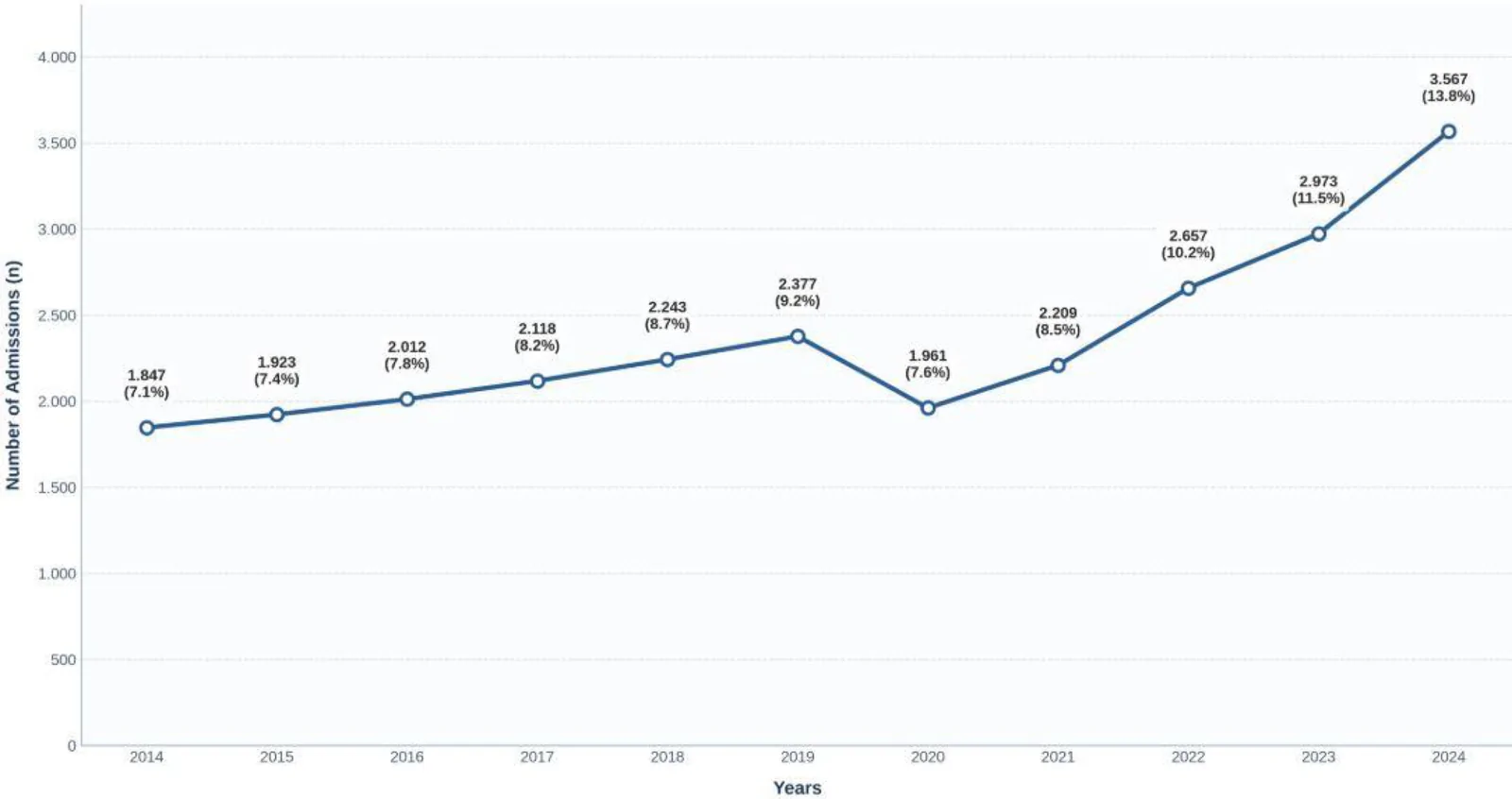
Distribution of forensic case presentations admitted to the emergency department by years.

**Table 1.** Baseline Demographic and Clinical Characteristics of Forensic Presentations (N = 25 887)

Variable	n	%
Age (years)		
Mean ± SD	34.0 ± 18.8	
Range (min-max)	1-97	
Age groups		
Pediatric (<18 years)	5223	20.2
Adult (≥18 years)	20 664	79.8
Gender		
Male	17 828	68.9
Female	8059	31.1
Mechanism of injury/reason for admission		
Motor vehicle accident	5435	21.0
Fall from height	4448	17.2
Assault	3283	12.7
Sharp/penetrating object injury	2995	11.6
Carbon monoxide poisoning	2836	11.0
Suicidal attempt	2257	8.7
Animal attack	1359	5.3
Firearm injury	819	3.2
Intoxication	760	2.9
Substance use	718	2.6
Burn	611	2.4
Electrocution	276	1.1
Sexual abuse	90	0.3
Total	25 887	100.0

**Table 2.** Treatment Modalities and Clinical Outcome Rates by Age Group

Variable		<18 years	≥18 years	*P*
Treatment type	Outpatient care	2162 (41.3%)	12 798 (61.9%)	<.001^*^
	Inpatient care	3061 (58.7%)	7866 (38.1%)	
Outcome	Death	109 (2.1%)	245 (1.2%)	<.001^*^
	Discharge	5114 (97.9%)	20 419 (98.8%)	

*Pearson chi-square test.

**Table 3.** Univariate and Multivariable Logistic Regression Analysis of Factors Associated with Mortality (N = 25 887)

Variables	Univariate OR (95% CI)	*P*	Adjusted OR (95% CI)*	*P*
Age (continuous)	1.05 (1.03-1.07)	<.001	1.04 (1.02-1.06)	**<.001**
Gender (male vs. female)	1.14 (1.01-1.28)	.041	1.08 (0.95-1.22)	.210
Month	1.01 (0.98-1.04)	.320	1.00 (0.97-1.03)	.850
ED-LOS (hours)	1.08 (1.04-1.13)	<.001	1.10 (1.05-1.16)	**<.001**
Mechanism of presentation				
Assault (reference)	**1.00 (Ref)**	**—**	**1.00 (Ref)**	**—**
Firearm injury	7.85 (4.20-13.40)	<.001	7.12 (3.80-11.90)	**<.001**
Carbon monoxide poisoning	5.24 (3.15-8.20)	<.001	4.85 (2.95-7.60)	**<.001**
Fall from height	3.12 (1.95-4.85)	<.001	2.90 (1.75-4.50)	**<.001**
Burn injury	3.40 (2.10-5.30)	<.001	3.15 (1.90-4.95)	**<.001**
Electric shock	2.65 (1.30-5.10)	.006	2.38 (1.10-4.80)	**.015**
Sharp/penetrating object injury	2.15 (1.40-3.20)	.001	1.95 (1.25-2.95)	**.002**
Motor vehicle accident	1.88 (1.25-2.75)	.002	1.70 (1.10-2.50)	**.010**
Intoxication	1.42 (0.92-2.15)	.110	1.35 (0.85-2.05)	.180
Suicidal attempt	1.08 (0.70-1.60)	.680	1.02 (0.65-1.55)	.910
Animal attack	0.85 (0.40-1.80)	.650	0.80 (0.35-1.70)	.610
Substance use	1.55 (0.85-2.70)	.150	1.40 (0.75-2.50)	.280
Sexual assault	0.92 (0.35-2.30)	.860	0.88 (0.30-2.20)	.820
Treatment type** (Ref: Inpatient care)**	5.10 (3.65-7.05)	<.001	4.65 (3.25-6.55)	**<.001**

Hosmer–Lemeshow test: *P* = .684 (model indicates a strong fit to the data).

OR, odds ratio; ED-LOS, length of stay in the emergency department; Ref, reference category.

*Adjusted for all variables in the table.

## Data Availability

The data that support the findings of this study are available on request from the corresponding author.
